# Editorial for the Special Issue “Cardiothoracic Imaging: Recent Techniques and Applications in Diagnostics”

**DOI:** 10.3390/diagnostics14050461

**Published:** 2024-02-20

**Authors:** Giacomo Sica, Gaetano Rea, Mariano Scaglione

**Affiliations:** 1 Department of Radiology, Azienda Ospedaliera dei Colli, Monaldi Hospital, 80131 Naples, Italy; gaetano.rea71@gmail.com; 2 Department of Medicine, Surgery and Pharmacy, University of Sassari, 07100 Sassari, Italy; mscaglione@uniss.it

Technology is making giant strides and is increasingly improving the diagnostic imaging of both frequent and rare acute and chronic diseases.

New technological advancements offer increasingly innovative techniques and applications to medical imaging, impacting the following different phases of the diagnostic process:

1. The development of new hardware: The introduction of spectral imaging through increasingly high-performance equipment such as dual energy technology finds its maximum technological expression in its recent introduction into clinical practice with new photon-counting detectors offering 0.25 mm pixel sizes and achieving ultra-high-resolution imaging, a technological revolution that could also detect changes in the microsemiology of certain diseases such as interstitial lung disease, which are frequently indeterminate and often require invasive procedures such as biopsies for histological evaluation [1,2]. The introduction of digital PET/CT technology, the newest evolution of clinical PET, enables advanced molecular imaging, improving image quality and subcentimeter cancer lesion detection over the analog PET/CT, and supports new opportunities for personalized nuclear medicine, enabling acquisition times to be shortened or radiotracer doses to be reduced [3], in addition to the possibility of reducing exam costs, and above all, the radiation exposure of patients.
2. The formulation of new clinical protocols: This is considered in light of the introduction of new equipment increasingly oriented toward saving radiation doses for patients [4].
3. Analysis of diagnostic images: Through the enormous potential of radiomics, this can be achieved by a new frontier of medicine based on the extraction of quantitative data from datasets of morphological and functional images of CT or PET/CT exams that cannot be detected by the human eye; the use of these data aims to create clinical decision support systems to improve non-invasive diagnoses, as well as improving therapies by making them increasingly tailored. Moreover, a quantitative approach toward radiomics could be useful to overcome some limitations of diagnostic imaging such as, for example, readers’ subjectiveness and the lack of repeatability in coronary computed tomography angiography and magnetic resonance imaging in the evaluation of cardiovascular diseases [5,6].
4. The introduction of artificial intelligence and machine learning algorithms applied to the development of postprocessing software: To date, this is the real hot topic in which most of the efforts toward technological development converge and which promises to facilitate increasingly accurate and early diagnoses, potentially also being able to solve further problems such as cutting waiting times for patients.

The concept of this Special Issue was to propose some of these important innovations for diagnostic imaging in cardiothoracic disorders, a group of diseases with a huge impact on mortality and the cost of medical care. In fact, cardiovascular and lung diseases are the leading cause of global deaths and one of the most serious health problems worldwide; they significantly increase the cost of medical care [7,8]. For example, lung cancer is the leading cause of cancer-related deaths, accounting for 18% of all cancer-related deaths worldwide and with over 2.2 million new cases being reported in 2020 [9]. Chronic obstructive pulmonary disease (COPD), the third cause of death worldwide, is frequently associated with a large number of comorbidities that contribute substantially to its poor outcome [10,11,12]. Heart and lung diseases frequently result in combination with each other, exerting nefarious synergistic power over each other, making life expectancy very low. Therefore, medical imaging might be helpful to personalize risk assessment, even in an asymptomatic preclinical stage, and therefore reducing mortality through the detection and early treatment of this group of diseases.

This Special Issue deals with purely clinical topics and other topics that introduce possible tools for future clinical practice, such as, for example, the definition of CT biomarkers in cardiogenic shock or the application of artificial intelligence in the diagnostic process of interstitial lung disease, which are often challenging entities for which high-resolution CT alone has always represented the diagnostic imaging gold standard, especially in hospitals not equipped with multidisciplinary teams.

When considering technological advancements, we must also mention the evolution of magnetic resonance imaging. For example, significant advancements have been made to cardiac MRI in the last decade; it has become the one-stop-shop tool in the diagnostic workup of various cardiac diseases [13,14]. In addition, recent technological developments, with strain techniques and the aid of advanced post-processing software have opened a huge chapter of study concerning the cardiotoxicity of oncological therapies, allowing the myriad of adverse cardiovascular effects associated with antineoplastic therapy to be detected [15,16].

Continuous technological improvements are also being made in hybrid and multimodality imaging such as PET/CT, PET/MR, and SPECT, improving the diagnostic performance of nuclear imaging and creating new possibilities in the early detection and diagnosis of cardiothoracic diseases, better clinical decisions, outcome prediction, or prognosis evaluation [17,18].

Finally, as mentioned before, technological evolution must include the development of tools capable of increasingly reducing the risk to patients in terms of reductions in exposure dose and the use of the iodinated contrast medium, which may have adverse effects and is time-consuming and costly, through the use of hardware equipment (dual-energy scanners) or through advanced, not-yet clinically validated tools of generative artificial intelligence that, for example, can avoid the use of iodinated contrast medium in angiographic CT examinations [19,20].

Therefore, to date, through technological progress applications in diagnostic imaging have reached very high levels of accuracy, and it is difficult to think about what the future still holds in this field. Certainly, however, public and private healthcare and all involved stakeholders will have to exert maximum effort so that all these present and future innovative diagnostic and prognostic tools can be accessible to all in order to progressively improve the diagnosis, and consequently, the therapy, of all cardiothoracic diseases by improving the quality of patients’ lives and reducing their mortality.

## References

[1] Si-Mohamed S.A., Miailhes J., Rodesch P.-A., Boccalini S., Lacombe H., Leitman V., Cottin V., Douek P. (2021). Spectral Photon-Counting CT Technology in Chest Imaging. J. Clin. Med..

[2] Remy-Jardin M., Hutt A., Flohr T., Faivre J.B., Felloni P., Khung S., Remy J. (2023). Ultra-High-Resolution Photon-Counting CT Imaging of the Chest A New Era for Morphology and Function. Investig. Radiol..

[3] Schillaci O., Urbano N. (2019). Digital PET/CT: A new intriguing chance for clinical nuclear medicine and personalized molecular imaging. Eur. J. Nucl. Med. Mol. Imaging.

[4] Rajendran K., Koo C.W. (2023). Photon-counting detector CT: Improving interstitial lung disease classification using ultra-high resolution at a fraction of the radiation dose?. Eur. Radiol..

[5] Polidori T., De Santis D., Rucci C., Tremamunno G., Piccinni G., Pugliese L., Zerunian M., Guido G., Pucciarelli F., Bracci B. (2023). Radiomics applications in cardiac imaging: A comprehensive review. Radiol. Med..

[6] Bakr S., Gevaert O., Echegaray S., Ayers K., Zhou M., Shafiq M., Zheng H., Benson J.A., Zhang W., Leung A.N.C. (2018). A radiogenomic dataset of non-small cell lung cancer. Sci. Data.

[7] Amini M., Zayeri F., Salehi M. (2021). Trend analysis of cardiovascular disease mortality, incidence, and mortality-to-incidence ratio: Results from global burden of disease study 2017. BMC Public Health.

[8] Vaduganathan M., Mensah G.A., Turco J.V., Fuster V., Roth G.A. (2022). The Global Burden of Cardiovascular Diseases and Risk: A Compass for Future Health. J. Am. Coll. Cardiol..

[9] International Agency for Research on Cancer World Health Organization. https://www.who.int/news-room/fact-sheets/detail/cancer.

[10] World Health Organization (2002). Global Initiative for Chronic Obstructive Lung Disease.

[11] Agusti A., Calverley P.M., Celli B., Coxson H.O., Edwards L.D., Lomas D.A. (2010). Characterization of COPD heterogeneity in the ECLIPSE cohort. Respir. Res..

[12] Divo M., Cote C., de Torres J.P., Casanova C., Marin J.M., Pinto-Plata V., Zulueta J., Cabrera C., Zagaceta J., Hunninghake G. (2012). Cocomorbidities and risk of mortality in patients with chronic obstructive pulmonary disease. Am. J. Respir Crit. Care Med..

[13] Blackwell G.G., Pohost G.M. (1995). The evolving role of MRI in the assessment of coronary artery disease. Am. J. Cardiol..

[14] Kramer C.M. (1998). Integrated approach to ischemic heart disease. The one-stop shop. Cardiol. Clin..

[15] Baldassarre L.A., Ganatra S., Lopez-Mattei J., Yang E.H., Zaha V.G., Wong T.C., Ayoub C., DeCara J.M., Dent S., Deswal A. (2022). Advances in Multimodality Imaging in Cardio-Oncology: JACC State-of-the-Art Review. J. Am. Coll. Cardiol..

[16] Jeong D., Gladish G., Chitiboi T., Fradley M.G., Gage K.L., Schiebler M.L. (2019). MRI in cardio-oncology: A review of cardiac complications in oncologic care. J. Magn. Reason. Imaging.

[17] Slart R.H.J.A., Williams M.C., Juarez-Orozco L.E., Rischpler C., Dweck M.R., Glaudemans A.W.J.M., Gimelli A., Georgoulias P., Gheysens O., Gaemperli O. (2021). Position paper of the EACVI and EANM on artificial intelligence applications in multimodality cardiovascular imaging using SPECT/CT, PET/CT, and cardiac CT. Eur. J. Nucl. Med. Mol. Imaging.

[18] Caobelli F., Cabrero J.B., Galea N., Haaf P., Loewe C., Luetkens J.A., Muscogiuri G., Francone M. (2023). Cardiovascular magnetic resonance (CMR) and positron emission tomography (PET) imaging in the diagnosis and follow-up of patients with acute myocarditis and chronic inflammatory cardiomyopathy: A review paper with practical recommendations on behalf of the Europeopean Society of Cardiovascular Radiology (ESCR). Int. J. Cardiovasc. Imaging.

[19] Gupta A., Kikano E.G., Bera K., Baruah D., Saboo S.S., Lennartz S., Hokamp N.G., Gholamrezanezhad A., Gilkeson R.C., Laukamp K.R. (2021). Dual energy imaging in cardiothoracic pathologies: A primer for radiologists and clinicians. Eur. J. Radiol Open.

[20] Lyu J., Fu Y., Yang M., Xiong Y., Duan Q., Duan C., Wang X., Xing X., Zhang D., Lin J. (2023). Generative Adversarial Network–based Noncontrast CT Angiography for Aorta and Carotid Arteries. Radiology.

